# Radiomics analysis of multiparametric MRI for preoperative prediction of microsatellite instability status in endometrial cancer: a dual-center study

**DOI:** 10.3389/fonc.2024.1333020

**Published:** 2024-01-29

**Authors:** Yaju Jia, Lina Hou, Jintao Zhao, Jialiang Ren, Dandan Li, Haiming Li, Yanfen Cui

**Affiliations:** ^1^ Department of Radiology, Shanxi Province Cancer Hospital/ Shanxi Hospital Affiliated to Cancer Hospital, Chinese Academy of Medical Sciences/Cancer Hospital Affiliated to Shanxi Medical University, Taiyuan, China; ^2^ Department of Radiology, Shanxi Traditional Chinese Medical Hospital, Taiyuan, China; ^3^ Department of Pharmaceuticals Diagnostics, GE HealthCare, Beijing, China; ^4^ Department of Radiology, Fudan University Shanghai Cancer Center, Shanghai, China; ^5^ Department of Oncology, Shanghai Medical College, Fudan University, Shanghai, China

**Keywords:** microsatellite instability, magnetic resonance imaging, radiomics, endometrial neoplasms, adjuvant therapy (post-operative)

## Abstract

**Objective:**

To develop and validate a multiparametric MRI-based radiomics model for prediction of microsatellite instability (MSI) status in patients with endometrial cancer (EC).

**Methods:**

A total of 225 patients from Center I including 158 in the training cohort and 67 in the internal testing cohort, and 132 patients from Center II were included as an external validation cohort. All the patients were pathologically confirmed EC who underwent pelvic MRI before treatment. The MSI status was confirmed by immunohistochemistry (IHC) staining. A total of 4245 features were extracted from T2-weighted imaging (T2WI), contrast enhanced T1-weighted imaging (CE-T1WI) and apparent diffusion coefficient (ADC) maps for each patient. Four feature selection steps were used, and then five machine learning models, including Logistic Regression (LR), k-Nearest Neighbors (KNN), Naive Bayes (NB), Support Vector Machine (SVM), and Random Forest (RF), were built for MSI status prediction in the training cohort. Receiver operating characteristics (ROC) curve and decision curve analysis (DCA) were used to evaluate the performance of these models.

**Results:**

The SVM model showed the best performance with an AUC of 0.905 (95%CI, 0.848-0.961) in the training cohort, and was subsequently validated in the internal testing cohort and external validation cohort, with the corresponding AUCs of 0.875 (95%CI, 0.762-0.988) and 0.862 (95%CI, 0.781-0.942), respectively. The DCA curve demonstrated favorable clinical utility.

**Conclusion:**

We developed and validated a multiparametric MRI-based radiomics model with gratifying performance in predicting MSI status, and could potentially be used to facilitate the decision-making on clinical treatment options in patients with EC.

## Introduction

Endometrial cancer (EC) is the sixth most common cancer in women worldwide, with approximately 417000 new cases in 2020, and the incidence is steadily increasing every year (1, 2). Microsatellite instability (MSI), a kind of deficient DNA mismatch repair (dMMR) system gene germline mutation, accounts for 20-30% in EC (3, 4). More than 90% of EC patients with dMMR have endometrioid adenocarcinoma (5). MSI status has been given increasing importance due to its value in terms of prognosis and treatment strategies for EC patients (4, 6). It has been reported that EC patients with high MSI (MSI-H) may be potential beneficiaries of immunotherapy response (7). Moreover, women with Lynch syndrome (LS) possess inherited pathogenic variants of MMR genes (6), and have a 40-60% risk of progressing to EC (8, 9). Therefore, MSI testing was recommended by the 2020 ESGO/ESTRO/ESP guidelines for all EC patients, which integrated MMR status for risk stratification of EC patients to assess prognosis and determine adjuvant therapy (10).

Currently, MSI status is identified by immunohistochemistry (IHC) for MMR protein expression or polymerase chain reaction (PCR) methods on biopsy or surgical tissue. However, both of these tissue sample-based methods are invasive and may not be sufficient to reflect the tumor heterogeneity. Furthermore, the PCR method is more costly and complicated due to the special equipment and reagents. Thus, exploring a non-invasive, cost-effective, and repeatable method to preoperatively predict MSI status will be helpful in clinical decision-making.

Pelvic magnetic resonance imaging (MRI) is recognized as a valuable imaging modality for the preoperative staging of EC in clinical practice (11). However, it is challenging to detect MSI status based on the subjective evaluation of MRI. Several studies have attempted to identify MSI status using functional imaging such as intravoxel incoherent motion (IVIM) and amide proton transfer (APT)-weighted imaging (12, 13). However, these functional imaging sequences have limited use in clinical settings due to their long scanning times and technical differences. Radiomics, an emerging tool for transforming digital medical images into mineable qualitative information, aims to reveal tumor heterogeneity and underlying pathophysiological information (14). Radiomics features extracted from MRI have been shown to predict risk stratification, prognosis, and treatment response in patients with EC (15–17). Several studies developed radiomics models to predict MSI status in patients with EC and achieved moderate performance (18, 19). However, these studies had a small sample size and lacked external validation.

Therefore, we attempted to develop and validate a non-invasive biomarker-based radiomics models from MRI of two centers to preoperatively evaluate the MSI status in patients with EC.

## Materials and methods

### Patients

**Figure 1:**
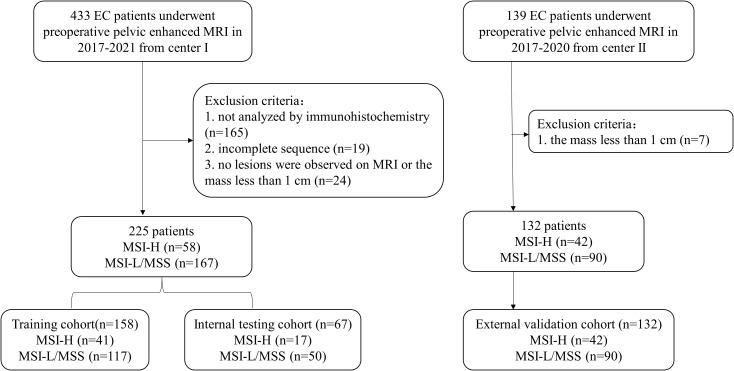
Flowchart of patients’ recruitment in Two Centers. EC, endometrial cancer; MSI-H, high microsatellite instability; MSI-L, low microsatellite instability; MSS, microsatellite stability.

This retrospective study was approved by the institutional review boards of the two participating centers, and the written informed consent was waived. A total of 225 patients with EC who underwent pelvic MRI between January 2017 and December 2021 at Center I (median age, 54.0 years) and 132 patients between January 2017 and December 2020 at Center II (median age, 54.0 years) were recruited (**Figure 1**). The recruitment criteria are provided in Appendix E1. Patients from Center I were randomly divided into two cohorts at a ratio of 7:3, with 158 cases in the training cohort and 67 cases in the internal testing cohort, and the patients from Center II served as the external validation cohort. Data regarding demographics and clinicopathological variables, including age, menopausal status, hypertension, diabetes, body mass index (BMI), the International Federation of Gynecology and Obstetrics (FIGO) stage, tumor grade, and depth of muscular invasion (MI), were collected from the medical records.

### MSI status identification

IHC for MMR proteins, including mutL homologue 1 (MLH1), mutS homologue 2 (MSH2), mutS homologue 6 (MSH6), and postmeiotic segregation increased 2 (PMS2), was performed to observe their expression in the nucleus. MLH1, PMS2, MSH2 and MSH6 were localized in the nucleus, and the presence of brownish-yellow granules in the nucleus of tumor cells was positive, and normal tissue staining was used as an internal control. In the nucleus of the tumor cells, all four MMR proteins show IHC staining is defined as the microsatellite stability (MSS)/low MSI (MSI-L), otherwise if one or more proteins are lost, the tumor is considered MSI-H (3).

### MRI acquisition

Preoperative pelvic MRI images for all patients at both institutions were acquired by 3.0T scanners. The MRI protocols included axial fat-suppressed spin-echo T2-weighted imaging (T2WI), echo planar diffusion-weighted imaging (DWI; with b value of 0, 800 s/mm2), and axial contrast enhanced T1-weighted imaging (CE-T1WI). Apparent diffusion coefficient (ADC) maps were generated automatically by DWI images using both b values. The imaging acquisition parameters are provided in Appendix E2.

### Tumor segmentation and radiomics features extraction

**Figure 2:**
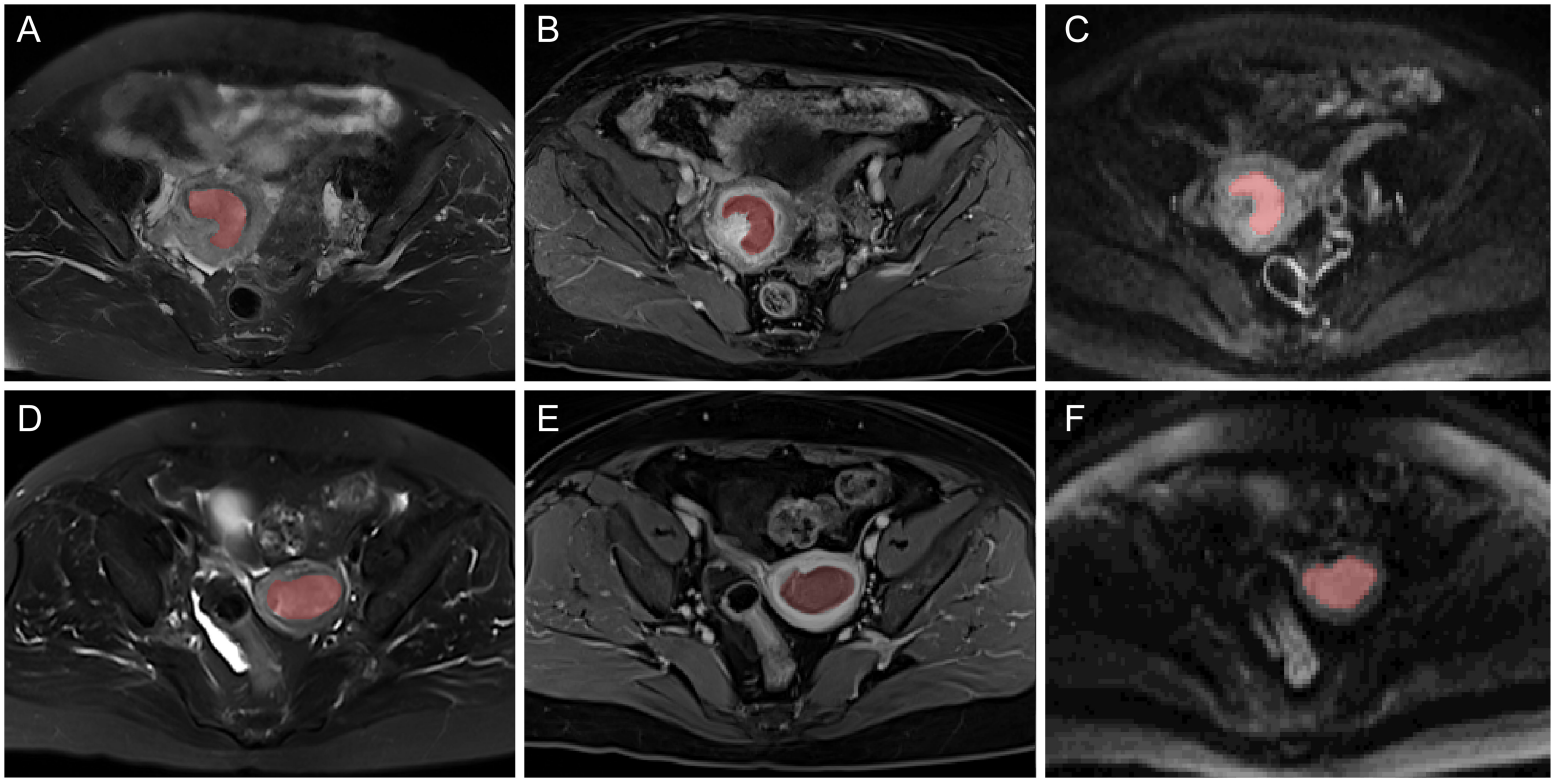
Typical multiparametric MR images of two EC patients with MSI-H **(A–C)** and MSI-L/MSS **(D–F)**. From left to right: axial T_2_WI, CE-T_1_WI, and DWI MR images and the corresponding region of interest (ROI).

MRI images, including axial T2WI fat-suppressed, CE-T1WI, and DWI were reevaluated by a radiologist with 10 years of pelvic MRI interpretation experience (**Figure 2**). Regions of interests (ROIs) of tumors were outlined manually layer-by-layer utilizing the ITK-SNAP software (version 3.8.0, http://www.itksnap.org), and volume of interests (VOI) were generated. The VOI outlined on the DWI automatically corresponds to the ADC map. Afterwards, 50 cases were randomly selected and outlined again by a physician with 15 years of experience in pelvic MRI interpretation, who was also blinded to the pathological data. The z-score method was used to normalize the grayscale of images, which was limited to *μ ± 3σ* (*μ*, mean value of gray levels; *σ*, gray level standard deviation).

Thereafter, the radiomics features were automatically extracted using PyRadiomics (version 3.0.1, http://PyRadiomics.readthedocs.io/en/latest/) (20). Finally, 1415 features were extracted from each sequence, including four types: (1) 14 shape features; (2) 18 first-order features; (3) 75 texture features such as GLCM, GLRLM, GLSZM, GLDM and NGTDM; (4) 1308 high-order features including 279, 744, 93, and 192 features derived from Laplacian of Gaussian (LoG), wavelet, gradient, and LocalBinaryPattern3D (LBP3D), respectively.

### Feature selection and modeling

We applied four steps to select radiomics features that were most related to MSI status. Firstly, to ensure the reproducibility of radiomics features, we calculated the intraclass correlation coefficient (ICC) to preserve features with good stability, and the ICC greater than 0.8 indicated good consistency. Secondly, based on univariate analysis, features with p<0.01 were preserved. Thirdly, the Pearson’s correlation analysis was performed to remove features with correlation coefficients greater than 0.9. Lastly, the Boruta algorithm (21) was used to retain the final residual features.

Five radiomics models based on different machine learning algorithms, including Logistic Regression (LR), k-Nearest Neighbors (KNN), Naive Bayes (NB), Support Vector Machine (SVM), and Random Forest (RF), were constructed using the selected key features. The predictive performance of the above models was evaluated based on the receiver operating characteristic (ROC) curve and area under the curve (AUC). The corresponding accuracy, sensitivity, specificity, negative prediction value and positive prediction value of the models were also obtained. The predictive performance of the five radiomics models was verified in the internal testing and external validation cohorts. Decision curve analysis (DCA) was employed to determine the clinical usefulness of the model by calculating the net benefit of the five different models at diverse threshold probabilities.

### Statistical analysis

All statistical analysis was conducted with the SPSS 25.0 software (IBM Corp) and R 4.1.0 software (http://www.Rproject.org, Appendix E3). The Chi-square test or Fisher’s exact test was used for categorical variables. Continuous data were selected from independent samples t-test or Mann-Whitney U-test depending on whether they conformed to normal distribution. The optimal cut-off value in the ROC was obtained according to the Youden’s index, and the differences in AUCs between the five models was compared using the Delong test. All statistical tests were two-sided, and P<0.05 was considered as statistically significant.

## Results

### Patient characteristics

The incidence of MSI-H was 26.0%, 25.4%, and 31.8% in the training cohort, the internal testing and external validation cohorts, respectively, with no statistical difference between the three cohorts (P=0.469). The patients from Center I had a slightly higher BMI and tumor grade than those from Center II, while other clinicopathological variables including age, menopausal status, hypertension, diabetes, the FIGO stage, and MI were not statistically significant (**Table 1**). There was no statistical difference between the MSI-H and MSI-L/MSS groups in terms of the above clinicopathological variables (**Supplementary Table S1**).

**Table 1:** Clinicopathological variables in patients with endometrial cancer between the training cohort and the internal and external validation cohorts.

	Training cohort (n=158)	Internal Testing cohort (n=67)	External Validation cohort (n=132)	*p* value
Age (y), median (IQR)	54.5 (50.0;59.0)	54.0 (50.0;59.0)	54.0 (50.0;59.3)	0.962
Label:				0.469
MSI-L/MSS	117 (74.1%)	50 (74.6%)	90 (68.2%)	
MSI-H	41 (25.9%)	17 (25.4%)	42 (31.8%)	
Menopause:				0.266
No	59 (37.3%)	20 (29.9%)	55 (41.7%)	
Yes	99 (62.7%)	47 (70.1%)	77 (58.3%)	
Hypertension:				0.941
Negative	94 (59.5%)	41 (61.2%)	81 (61.4%)	
Positive	64 (40.5%)	26 (38.8%)	51 (38.6%)	
Diabetes:				0.456
Negative	141 (89.2%)	63 (94.0%)	117 (88.6%)	
Positive	17 (10.8%)	4 (6.0%)	15 (11.4%)	
BMI, median (IQR)	26.7 (24.1;29.0)	26.0 (24.5;28.5)	24.4 (22.6;27.3)	<0.001*
FIGO:				0.404
Ia	92 (58.2%)	38 (56.7%)	80 (60.6%)	
Ib	45 (28.5%)	19 (28.4%)	25 (18.9%)	
II	14 (8.9%)	8 (11.9%)	20 (15.2%)	
III	7 (4.4%)	2 (3.0%)	7 (5.3%)	
Grade:				<0.001*
1	17 (11.2%)	6 (9. 7%)	63 (47.7%)	
2	116 (76.3%)	46 (74.2%)	54 (40.9%)	
3	19 (12.5%)	10 (16.1%)	15 (11.4%)	
MI:				0.477
<1/2	108 (68.4%)	45 (67.2%)	97 (74.0%)	
≥1/2	50 (31.6%)	22 (32.8%)	34 (26.0%)	

BMI, body mass index; FIGO, the International Federation of Gynecology and Obstetrics; MI, muscular invasion.

*p <0.05, suggest a significant difference between the three cohorts.

### Feature selection and prediction model construction

**Figure 3:**
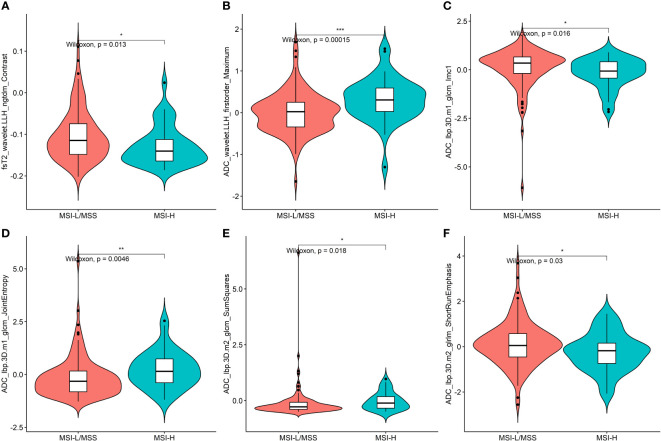
Plots **(A–F)** present the boxplots of the six radiomics features with a significant difference between the MSI-H and MSI-L/MSS groups in the training cohort. * = p < 0.5; ** = p < 0.01; *** = p < 0.001.

Of all the extracted features, 1464 features with high reproducibility (ICC>0.8) were selected. Moreover, 252 features were retained after univariate analysis, and 66 related features were selected by Pearson’s correlation analysis. Six key features, found to be most relevant to MSI status, were screened using the Boruta algorithm, which was then used for the construction of the radiomics model. The selected six features, including first order feature, GLCM, GLRLM, and NGTDM, were statistically different between the MSI-H and MSI-L/MSS groups (all P < 0.05) (**Figure 3**).

### Performance and validation of prediction models

**Figure 4:**
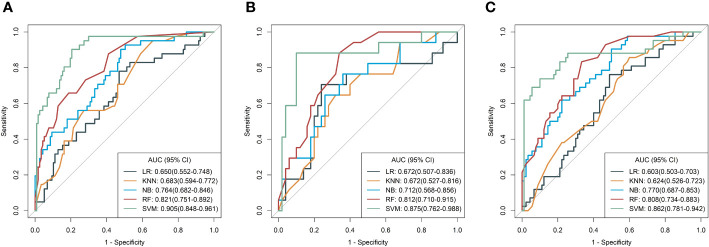
Receiver operating characteristic (ROC) curves of the radiomics models derived from five classifiers in the training **(A)**, internal test **(B)**, and external validation **(C)** cohorts, respectively.

**Figure 5:**
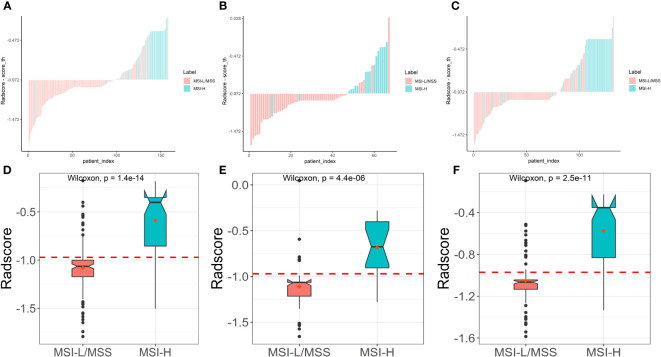
Plots **(A–C)** show the Rad-score for each patient, and boxplots **(D–F)** show the Rad-score in the training, internal test and external validation cohorts, respectively.

Based on five different classifiers such as LR, KNN, NB, SVM, and RF, the radiomics models were constructed in the training cohort using the above six key features for prediction of MSI status. The ROC curves depicting the predictive performance of these different models are shown in **Figure 4**; **Table 2**. The SVM model showed the best performance for prediction of MSI status in the training cohort, internal testing cohort and external validation cohort with AUCs of 0.905 (95% confidence interval [CI], 0.848-0.961), 0.875 (95%CI, 0.762-0.988), and 0.862 (95%CI, 0.781-0.942), respectively. The Rad-scores derived from the SVM model of the MSI-H group were significantly higher than those of the MSI-L/MSS group in the training cohort (**Figure 5A, D**), and confirmed in both validation cohorts (**Figure 5B, C, E, F**). **Table 2** shows the performance differences between the SVM model and others.

**Table 2:** Performance of the radiomics models.

	Method	LR	KNN	NB	RF	SVM
**Training cohort**	AUC (95%CI)	0.650 (0.552-0.748)	0.683 (0.594-0.772)	0.764 (0.682-0.846)	0.821 (0.751-0.892)	0.905 (0.848-0.961)
	Accuracy (95%CI)	0.595 (0.514-0.672)	0.500 (0.420-0.580)	0.620 (0.540-0.696)	0.772 (0.699-0.835)	0.823 (0.754-0.879)
	Sensitivity (95%CI)	0.780 (0.487-0.927)	0.951 (0.820-1.000)	0.902 (0.659-0.976)	0.659(0.484-0.805)	0.902 (0.731-0.976)
	specificity (95%CI)	0.530 (0.213-0.624)	0.342 (0.141-0.462)	0.521 (0.248-0.624)	0.812 (0.587-0.920)	0.795 (0.667-0.881)
**Internal Testing cohort**	AUC (95%CI)	0.672 (0.507-0.836)	0.672 (0.527-0.816)	0.712 (0.568-0.856)	0.812 (0.710-0.915)	0.875 (0.762-0.988)
	Accuracy (95%CI)	0.582 (0.455-0.702)	0.478 (0.354-0.603)	0.537 (0.411-0.660)	0.701 (0.577-0.807)	0.896 (0.797-0.957)
	Sensitivity (95%CI)	0.765 (0.588-0.941)	0.941 (0.647-1.000)	0.824 (0.646-1.000)	0.765 (0.529-1.000)	0.882 (0.412-1.000)
	Specificity (95%CI)	0.520 (0.080-0.861)	0.320(0.080-0.621)	0.440 (0.200-0.740)	0.680 (0.530-0.880)	0.900 (0.160-0.961)
**External Validation cohort**	AUC (95%CI)	0.603 (0.503-0.703)	0.624 (0.526-0.723)	0.770 (0.687-0.853)	0.808 (0.734-0.883)	0.862 (0.781-0.942)
	Accuracy (95%CI)	0.439 (0.353-0.528)	0.432 (0.346-0.521)	0.530 (0.442-0.618)	0.720 (0.635-0.794)	0.795 (0.717-0.861)
	Sensitivity (95%CI)	0.857 (0.714-0.976)	0.952 (0.881-1.000)	0.976 (0.929-1.000)	0.833 (0.571-0.929)	0.810 (0.667-0.952)
	Specificity (95%CI)	0.244 (0.178-0.567)	0.189 (0.044-0.456)	0.322 (0.111-0.533)	0.667 (0.500-0.756)	0.789 (0.289-0.945)

AUC, area under ROC curve; CI, confidence interval; LR, Logistic Regression; KNN, k-Nearest Neighbors; NB, Naive Bayes; RF, Random Forest; SVM, Support Vector Machine.

### Clinical application

**Figure 6:**
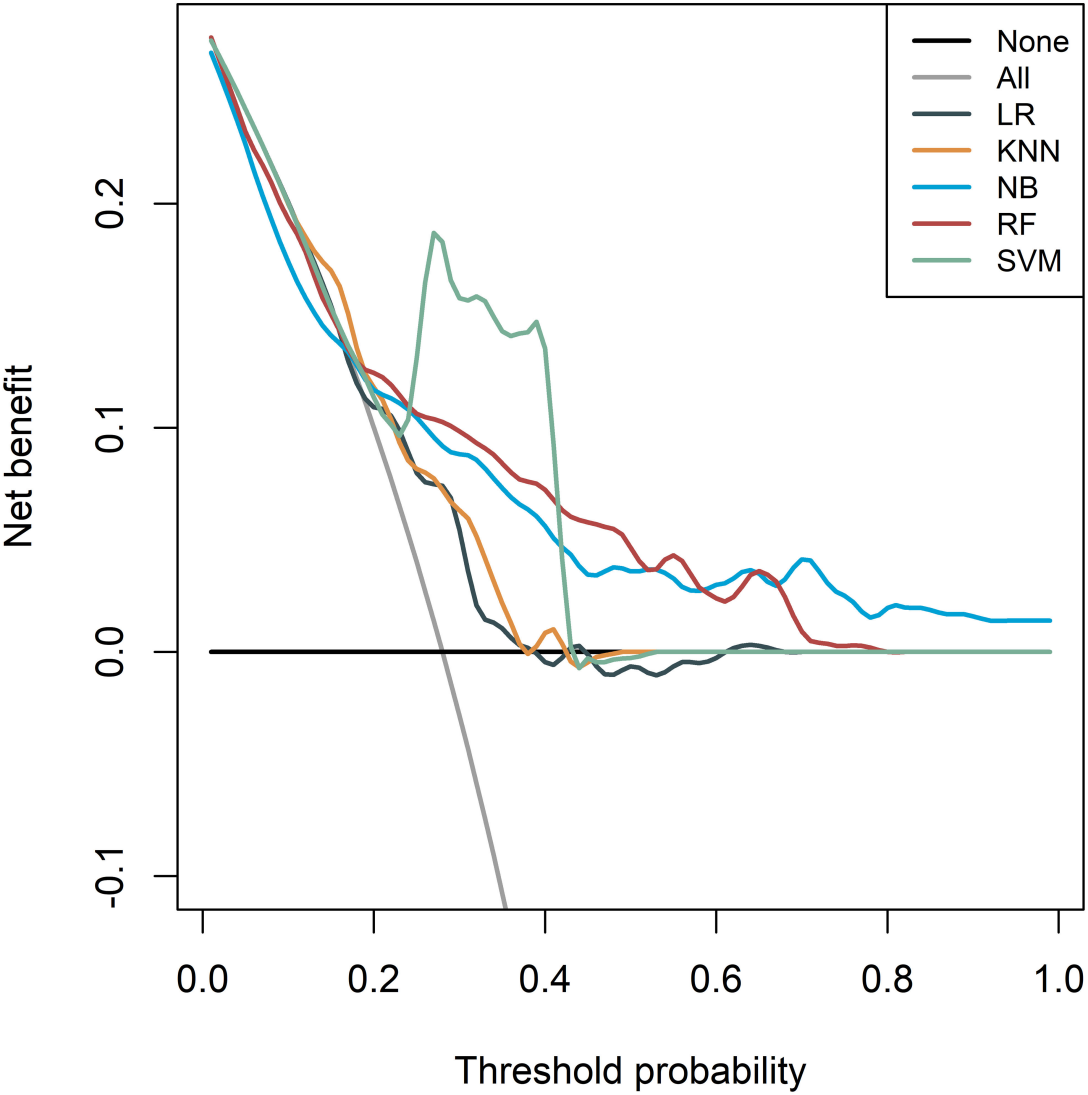
Decision curve analysis (DCA) of the radiomics models derived from the five different classifiers in the whole cohorts.

The DCA of different models is presented in **Figure 6**, which indicated that the SVM model had a higher net benefit compared to the other models, both in the training cohort and the two validation cohorts. Moreover, the net benefit of all models was better than that of diagnosing all patients as “MSI-H” or “MSI-L/MSS”. These results showed that the radiomics model had good clinical application value for preoperative evaluation of MSI status.

## Discussion

In this study, we developed radiomics models based on multiparametric MR images (T2WI, CE-T1WI, and ADC) to identify the MSI status of EC, which were validated in patients from two institutions. Among the models constructed based on five different machine learning algorithms, the SVM model showed the best prediction performance with an AUC of 0.905 and was subsequently verified in both the internal testing and external validation cohorts. The clinicopathological variables were not found to be related to MSI status. These results indicated that radiomics methods could be a non-invasive tool for prediction of MSI status preoperatively.

Our machine learning classifiers were built on six radiomic features, four of which were LBP transformed. LBP provided a binary label for each pixel value in the image based on a specific threshold calculated from the value of neighboring pixels around the center pixel, which can highlight the local texture features of the image (22, 23). Furthermore, higher-order features account for the largest proportion (5/6), which provide complex texture information to quantify tumor heterogeneity by emphasizing the relationships between multiple voxels (24). Notably, GLCM features accounted for half of the features, suggesting that GLCM may be more closely related to the MMR genes, which was consistent with the findings of a previous study (25), which found that GLCM entropy was different in patients with and without Lynch syndrome, which may be related to mutations in the MMR genes. GLCM features can obtain more information than histogram of an image, and represent the heterogeneity differences in the image in two dimensions.

The good predictive performance of the model is closely related to the choice of classifier. In this study, five different classifiers were used to detect the MSI status of EC. The results showed that the SVM model had the highest predictive performance. SVM is known for its ability to be robust to noise and to handle high-dimensional datasets in genetics, transcriptomics and proteomics. SVM has shown good performance in predicting lymph node metastasis of breast cancer and gene status in patients with rectal cancer (26, 27). A recent study also found that SVM was the best model to predict EC MSI (28). Therefore, it is not surprising that our study found that SVM is the best predictor of MSI status in EC patients.

Few studies have explored whether radiomics features derived from MRI are correlated with the MSI status of EC. A previous study analyzed 12 patients with stage I EC on reduced field of view DWI, and found that MSS tumors had a significantly higher ADC value than the MSI tumors, indicating that MRI may contain information related to MSI (12). However, whether the ADC value can be used to predict MSI status remains controversial. Minamiguchi et al. (29) and Wang et al. (30) found no difference in ADC values between the MSI and MSS groups. In our study, the majority (5/6) of the key radiomics features were derived from ADC maps, indicating that the information related to tumor heterogeneity embedded within ADC images cannot be reflected by simple ADC values alone. Recently, Veeraraghavan et al. demonstrated that a classifier based on CE-CT peritumoral-rim radiomics is the most relevant to distinguish dMMR of EC, with an AUC of 0.78 (18). However, CT has great challenges in delineating the tumor margins, whereas MRI may accurately depict tumor boundaries because of its better soft-tissue resolution. Lin et al. constructed a model based on T2WI and CE-T1WI of 296 patients to predict MSI of EC, with AUCs of 0.752 and 0.723 in the training cohort and validation cohorts (19). In our study, radiomics features were extracted from multiple sequences of MRI (T2WI, CE-T1WI, and ADC) and showed good performance, similar to a recent study by Song et al. (28).

Among the clinicopathological variables mentioned in the present study, no significant difference was found between the MSI-H and MSI-L/MSS groups, similar to the findings of a recent study (30). A large-scale study including 1024 patients with endometrioid EC found that age, BMI, and grade were associated with dMMR (31), which were not found in our study, possibly due to insufficient sample size.

There are several limitations to this study. Firstly, manual segmentation of ROI was not only labor-intensive but also tended to be subjective. Hence, if semi-automatic or automatic delineation can be used in the future, it will be more convenient in clinical practice. Secondly, this study was limited to primary tumors. Since the FDA approved pembrolizumab is more effective in patients with recurrent and metastatic disease, it will be necessary to validate our models in patients with advanced disease. Finally, this study did not consider the peritumoral region. Veeraraghavan et al. (18) suggested that the MMR status might be related to peritumoral region, which needs to be explored in the future.

In summary, we presented a non-invasive radiomics approach utilizing multiparametric MRI to identify MSI status in patients with EC prior to treatment. Our results suggested that radiomics approaches can potentially aid decision-making and maximize benefit for EC patients.

## Data availability statement

The raw data supporting the conclusions of this article will be made available by the authors, without undue reservation.

## Ethics statement

The studies involving humans were approved by Shanxi Province Cancer Hospital/Shanxi Hospital Affiliated to Cancer Hospital, Chinese Academy of Medical Sciences/Cancer Hospital Affiliated to Shanxi Medical University. The studies were conducted in accordance with the local legislation and institutional requirements. The ethics committee/institutional review board waived the requirement of written informed consent for participation from the participants or the participants’ legal guardians/next of kin because Written informed consent was waived by the Institutional Review Board.

## Author contributions

YJ: Data curation, Investigation, Writing – original draft. LH: Methodology, Project administration, Supervision, Writing – review & editing. JZ: Data curation, Investigation, Writing – original draft. JR: Formal analysis, Software, Visualization, Writing – original draft. DL: Conceptualization, Data curation, Investigation, Writing – original draft. HL: Data curation, Funding acquisition, Validation, Writing – review & editing. YC: Conceptualization, Funding acquisition, Writing – review & editing.
