# Supplementary material for: Radiomics analysis of multiparametric MRI for preoperative prediction of microsatellite instability status in endometrial cancer: a dual-center study
Source: Front Oncol. 2024 Jan 29;14:1333020. doi: 10.3389/fonc.2024.1333020 (PMC10860747; doi:10.3389/fonc.2024.1333020)
Supplement: Supplementary file 1 [file DataSheet_1.docx]

**Supplementary Materials**

**Appendix E1: Patient Recruitment**

The recruitment process met the following criteria: Inclusion criteria: (1) all patients underwent radical surgical resection; (2) the histological subtype of the patients is endometrioid adenocarcinoma; (3) no history of treatment before operation. Exclusion criteria: (1) surgical specimens were not analyzed by MMR protein immunohistochemistry; (2) poor image quality or incomplete sequence; (3) no lesions were observed on MRI or the mass less than 1 cm.

**Appendix E2 MR images acquisition parameters**

All patients from center I underwent pelvic MR examination before treatment with a 3.0T system (Discovery MR 750; GE Medical Systems, Milwaukee, Wis) equipped with an 8-channel phased array torso coils. The MRI protocol consisted of the following: (a) fat-suppressed spin-echo T2-weighted imaging (T2WI) was acquired with TR/TE: 7560/120 ms, FOV of 360 mm×360 mm, matrix of 400×400, NEX of 2, slice thickness of 5 mm, and slice gap=1 mm. (b) Axial diffusion weighted imaging (DWI) with b value of 0, 800 s/mm^2^ with TR/TE: 6200/71 ms, flip angle of 90°, FOV of 360 mm×360 mm, matrix of 128×128, NEX of 2, slice thickness of 5 mm, and slice gap=1 mm. Apparent diffusion coefficient (ADC) maps were generated automatically in a mono-exponential decay model using both two b values. (c) contrast enhanced T1-weighted imaging (cT1WI) was performed after intravenous administration of a 0.1 mmol/kg dose of Gd-DTPA (Magnevist; Bayer-Schering Pharma AG, Berlin, Germany). Scan parameters were TR/TE: 4.9/1.3 ms, flip angle of 15°, FOV of 380 mm×380 mm, matrix of 256×256, NEX of 1, slice thickness of 4 mm, and no slice gap.

The patients from Center II were examined using the 3.0T MR (Magnetom Skyra, Siemens Healthcare), with the acquisition parameters as following: (a) the axial fat-suppressed T2-weighted sequence was acquired with TR/TE: 3600/77 ms, FOV of 240×240 mm, average of 2, matrix of 320×192, flip angle of 166°, slice thickness of 5 mm, and slice gap of 1 mm. (b) Axial DWI with TR/TE: 9260/60 ms, average of 4, matrix of 320×240, flip angle of 90°, slice thickness of 5 mm, and slice gap of 0.4 mm. (c) The contrast-enhanced T1-weighted sequence (cT1WI) with TR/TE: 3.85/1.45 ms, FOV of 360 mm×360 mm, average of 3, matrix of 256×256, flip angle of 9°, slice thickness of 3 mm, and no slice gap.

***Abbreviations:*** TR: repetition time; TE: echo time; FOV: field of view; NEX: number of excitation.

**Appendix E3. The Statistical Analysis and Packages of R Software**

The ROC curves were plotted using the “pROC” package. LR, KNN, NB, SVM, and RF classifiers were based on the "base", “class”, “nb”, “kernlab”, “randomforest” package, respectively. The Hosmer-Lemeshow test was performed using the " Resource Selection " package. DCA was performed with the function of “dca.R”.

| **Appendix Table 1 Clinicopathological variables in patients with endometrial cancer between MSI-L/MSS cohorts and MSI-H cohorts** | | | | | | | | | |
| --- | --- | --- | --- | --- | --- | --- | --- | --- | --- |
| **variables** | **Training cohort n=158** | | ***p* value** | **Internal Testing cohort n=67** | | ***p* value** | **External Validation cohort n=132** | | ***p* value** |
|  | MSI-L/MSS  (n =117) | MSI-H  (n =41) |  | MSI-L/MSS  (n =50) | MSI-H  (n =17) |  | MSI-L/MSS  (n =90) | MSI-H  (n =42) |  |
| Age(y), median (IQR) | 54.0(49.0-59.0) | 56.0(52.0-60.0) | 0.115 | 54.0(50.0-59.0) | 55.0(51.0-58.0) | 0.885 | 54.0(50.0-60.8) | 52.5(50.0-57.0) | 0.248 |
| Menopause |  |  | 0.153 |  |  | 0.334 |  |  | 1.000 |
| No | 48 (41.0%) | 11 (26.8%) |  | 17 (34.0%) | 3 (17.6%) |  | 37 (41.1%) | 18 (42.9%) |  |
| Yes | 69 (59.0%) | 30 (73.2%) |  | 33 (66.0%) | 14 (82.4%) |  | 53 (58. 9%) | 24 (57.1%) |  |
| Hypertension |  |  | 0.436 |  |  | 0.955 |  |  | 0.507 |
| Negative | 67 (57.3%) | 27 (65.9%) |  | 30 (60.0%) | 11 (64.7%) |  | 53 (58. 9%) | 28 (66. 7%) |  |
| Positive | 50 (42.7%) | 14 (34.1%) |  | 20 (40.0%) | 6 (35.3%) |  | 37 (41.1%) | 14 (33.3%) |  |
| Diabetes |  |  | 1.000 |  |  | 0.565 |  |  | 0.143 |
| Negative | 104 (88.9%) | 37 (90.2%) |  | 46 (92.0%) | 17 (100.0%) |  | 77 (85.6%) | 40 (95.2%) |  |
| Positive | 13 (11.1%) | 4 (9.8%) |  | 4 (8.0%) | 0 (0.0%) |  | 13 (14.4%) | 2 (4.8%) |  |
| BMI, median (IQR) | 26.9(24.0-29.3) | 26.0(24.2-28.1) | 0.360 | 26.1(24.6-28.6) | 25.6(24.5-27.0) | 0.768 | 24.4(22.3-27.4) | 24.4(22.7-26.9) | 0.895 |
| FIGO |  |  | 0.677 |  |  | 0.133 |  |  | 0.625 |
| Ia | 70 (59.8%) | 22 (53.7%) |  | 28 (56.0%) | 10 (58.8%) |  | 56 (62.2%) | 24 (57.1%) |  |
| Ib | 32 (27.4%) | 13 (31.7%) |  | 15 (30.0%) | 4 (23.5%) |  | 15 (16. 7%) | 10 (23.8%) |  |
| II | 9 (7.7%) | 5 (12.2%) |  | 7 (14.0%) | 1 (5.9%) |  | 15 (16. 7%) | 5 (11.9%) |  |
| III | 6 (5.1%) | 1 (2.4%) |  | 0 (0.0%) | 2 (11.8%) |  | 4 (4.4%) | 3 (7.1%) |  |
| Grade |  |  | 0.120 |  |  | 1.000 |  |  | 0.140 |
| 1 | 15 (13.2%) | 2 (5.3%) |  | 5 (11.1%) | 1 (5.9%) |  | 48 (53.3%) | 15 (35.7%) |  |
| 2 | 88 (77.2%) | 28 (73.7%) |  | 33 (73.3%) | 13 (76.5%) |  | 32 (35. 6%) | 22 (52.4%) |  |
| 3 | 11 (9.6%) | 8 (21.0%) |  | 7 (15. 6%) | 3 (17.6%) |  | 10 (11.1%) | 5 (11.9%) |  |
| MI |  |  | 0.838 |  |  | 0.252 |  |  | 0.798 |
| ＜1/2 | 81 (69.2%) | 27 (65.9%) |  | 36 (72.0%) | 9 (52.9%) |  | 67 (75.3%) | 30 (71.4%) |  |
| ≥1/2 | 36 (30.8%) | 14 (34.1%) |  | 14 (28.0%) | 8 (47.1%) |  | 22 (24.7%) | 12 (28.6%) |  |
| **Abbreviations:** BMI, body mass index; FIGO, the International Federation of Gynecology and Obstetrics; MI, muscular invasion. | | | | | | | | | |
